# Case report: A case of benign uterine leiomyoma with intense ^18^F-FDG uptake mimicking malignant tumor on PET/CT

**DOI:** 10.3389/fonc.2026.1817471

**Published:** 2026-05-19

**Authors:** Taiping Liao, Guoxu Fu, Yongjun Long, Songsong Yang, Jun He, Jun Li

**Affiliations:** 1 Department of Nuclear Medicine, The Third Hospital of Mianyang(Sichuan Mental Health Center), Mianyang, China; 2 Department of Nuclear Medicine, Zigong Fourth People’s Hospital, Zigong, China; 3 Department of Oncology, The Third Hospital of Mianyang(Sichuan Mental Health Center), Mianyang, China

**Keywords:** ^18^F-FDG, case report, diagnostic pitfall, PET/CT, uterine leiomyoma

## Abstract

Uterine leiomyoma is a common benign tumor of the uterus and typically demonstrates low FDG uptake on ^18^F-FDG PET/CT. Intense FDG uptake in a uterine mass is generally considered suggestive of malignancy. Here, we report a case of uterine leiomyoma in a patient who presented with multiple areas of abnormal bone density throughout the body. To evaluate the possibility of bone metastases and to identify a potential primary tumor, ^18^F-FDG PET/CT was performed. Incidentally, a uterine mass with markedly increased FDG uptake was detected, raising strong suspicion of a uterine malignancy. However, postoperative histopathological examination ultimately confirmed the diagnosis of a benign uterine leiomyoma.

## Introduction

Uterine leiomyoma is the most common benign tumor of the female reproductive system. It typically originates from smooth muscle cells of the myometrium and is often detected incidentally during routine examinations or when patients present with gynecological symptoms (1). From an imaging perspective, uterine leiomyomas usually appear as well-circumscribed masses with characteristic morphological features. Accurate differentiation between benign leiomyomas and malignant uterine tumors is crucial for establishing appropriate clinical management strategies.

^18^F-FDG PET/CT plays an important role in oncologic imaging, particularly in tumor staging, evaluation of metastatic disease, and detection of occult primary malignancies (2). In general, benign uterine leiomyomas demonstrate absent or only mild FDG uptake (3), whereas malignant uterine tumors typically exhibit markedly increased glucose metabolism (4). However, there is a substantial overlap in FDG uptake between benign and malignant uterine lesions, and FDG avidity alone is not sufficiently specific for definitive characterization.

According to previous reports, a very small proportion of benign uterine leiomyomas may demonstrate intense FDG uptake (SUVmax range: 22.8-23.5), mimicking the imaging appearance of malignant uterine tumors on PET/CT (5, 6). This phenomenon represents an important diagnostic pitfall, as it may lead to false-positive interpretations and potentially unnecessary aggressive interventions. Although several studies have described this unusual PET/CT presentation of uterine leiomyomas, cases with extremely high FDG uptake remain relatively rare, and their imaging characteristics have not yet been fully elucidated.

Herein, we report a case of uterine leiomyoma that was incidentally identified during an ^18^F-FDG PET/CT examination performed for suspected bone metastases. This case is noteworthy for several distinctive features. First, one of the uterine lesions demonstrated exceptionally intense FDG uptake, with a very high SUVmax that further increased on delayed imaging, a pattern that is rarely reported in benign leiomyomas. Second, two uterine lesions within the same uterus exhibited markedly different metabolic characteristics, providing an internal comparison that further complicated interpretation. Third, the examination was performed in the clinical context of suspected metastatic disease, which increased the likelihood of misinterpreting the hypermetabolic uterine lesion as a primary malignancy. These combined features make this case particularly illustrative of a potential diagnostic pitfall. It highlights the limitations of relying solely on FDG metabolic information for differentiating benign from malignant uterine lesions and underscores the importance of comprehensive interpretation incorporating metabolic activity, morphological features, and clinical context.

## Case presentation

A 35-year-old woman was found to have a uterine mass during a routine physical examination five years earlier. Recently, due to 42 days of amenorrhea, she underwent ultrasonography, which revealed a marked increase in the size of the uterine mass compared with previous findings. The lesions in both the anterior and posterior uterine walls appeared hypoechoic, with a few linear blood flow signals observed at the margins and scattered punctate blood flow signals within the lesions. The patient is G1P1, with no history of hormonal medication or oral contraceptive use. The current pregnancy test was negative (HCG<0.5 mIU/mL). Aside from amenorrhea, the patient reports no abnormal uterine bleeding, pain, or other gynecologic symptoms. She was subsequently admitted for surgical treatment.

**Figure 1:**
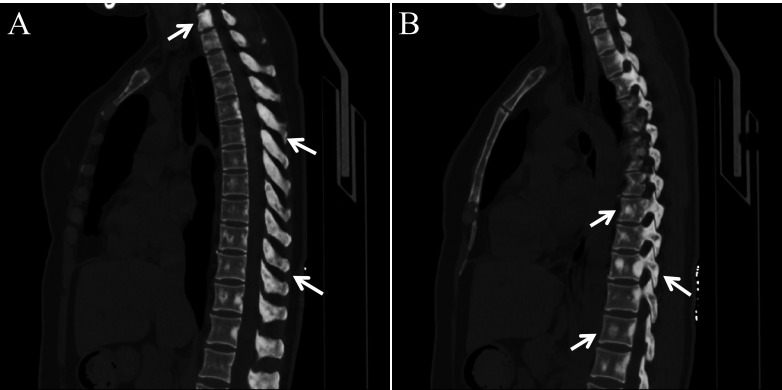
Sagittal chest computed tomography images **(A, B)** demonstrate multiple slightly hyperdense lesions in the patient’s spine (arrows).

**Figure 2:**
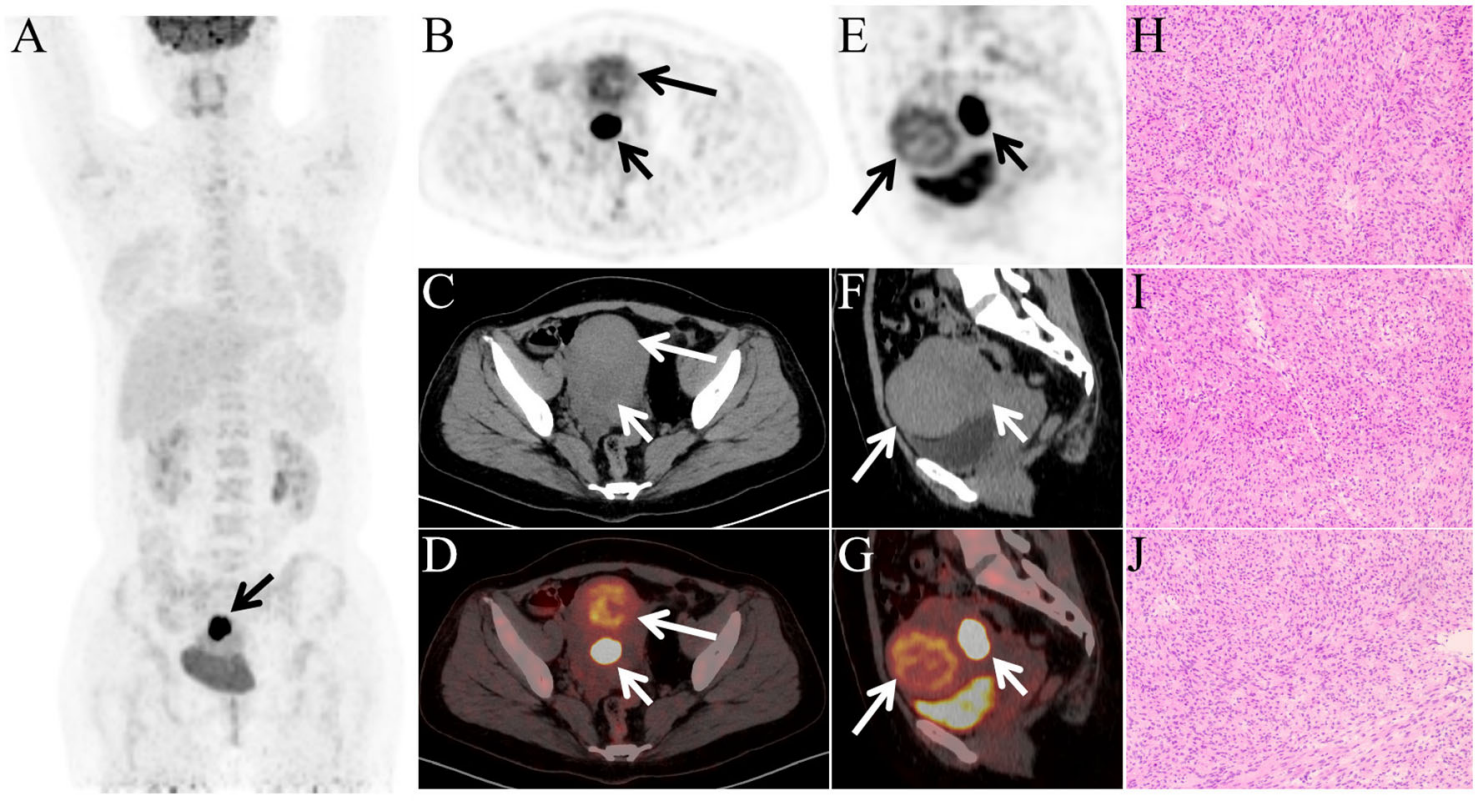
**(A)** Maximum intensity projection (MIP) image shows focal increased FDG uptake in the pelvic region (arrow). **(B–G)** Anterior lesion (long arrows) shows mild FDG uptake (SUVmax 5.8); posterior lesion (short arrows) exhibits marked uptake (SUVmax 26.5), increasing on delayed imaging (SUVmax 40.8). **(H–J)** Histopathological examination of the posterior uterine wall lesion (hematoxylin and eosin staining, ×100) demonstrates typical features of a benign leiomyoma, including uniform spindle-shaped smooth muscle cells arranged in interlacing fascicles, absence of cytologic atypia and tumor cell necrosis.

After admission, chest computed tomography revealed multiple slightly hyperdense nodules throughout the skeletal system, most prominently involving the spine (**Figures 1A, B**), raising suspicion of bone metastases. To determine whether these skeletal lesions represented metastatic disease, the patient underwent an ^18^F-FDG PET/CT examination. The PET/CT model used was the United Imaging uMI 550. The patient’s blood glucose level at the time of injection was 5.4 mmol/L. ^18^F-FDG was administered according to a standard dose of 3.7 MBq/kg.​ Image acquisition began 1 hour after radiotracer injection, and a delayed scan was performed 2 hours post-injection. Images were reconstructed using the ordered subset expectation maximization (OSEM) algorithm. The SUV values were calculated based on the patient’s Body Mass Index (BMI) using the formula:

$\text{SUV}=\frac{\text{Radioactivity concentration of the ROI (Bq/g)}}{{\text{Injected radioactivity (Bq)/BMI(kg/m}}^{2})}/k$, where ROI represents the region of interest, and k is the radionuclide decay correction factor. The PET/CT images (**Figures 2A–G**) showed no abnormally increased FDG uptake in the systemic skeletal lesions, suggesting that these lesions are more likely to be benign rather than metastatic. Notably, the mass located on the posterior wall of the uterus demonstrated extremely intense FDG uptake (SUVmax 26.5, delayed SUVmax 40.8), strongly suggesting the possibility of malignancy, such as uterine sarcoma or lymphoma.

Laboratory investigations, including liver and renal function tests, electrolyte levels, complete blood count, infection markers, and tumor markers, were all within normal limits. The patient subsequently underwent surgical resection of the uterine mass. The histopathological examination of the anterior uterine wall mass revealed no nuclear atypia, no abnormal mitotic figures, and no coagulative necrosis. The lesion was diagnosed as a conventional (usual-type) uterine leiomyoma. Histopathological examination of the posterior uterine wall lesion demonstrated a proliferation of uniform spindle-shaped smooth muscle cells arranged in interlacing fascicles (**Figures 2H–J**). No significant cytologic atypia or nuclear pleomorphism was identified. There was no evidence of coagulative tumor cell necrosis, hyaline degeneration, hemorrhage, cystic change, or red degeneration. Mitotic activity was low, with approximately 1 mitosis per 10 high-power fields. Based on these findings, the posterior uterine wall lesion was classified as a conventional leiomyoma rather than a special histologic variant. The absence of atypia, tumor cell necrosis, and increased mitotic activity effectively excludes the diagnosis of leiomyosarcoma and smooth muscle tumor of uncertain malignant potential (STUMP). The patient’s disease timeline is presented in **Table 1**.

**Table 1:** Timeline of clinical presentation and management.

Date	Event
2020/8/2	A hypoechoic uterine nodule (1.3cm × 1.1cm) was detected on routine health check-up by ultrasonography.
2022/6/3	A follow-up ultrasound showed enlargement of the anterior uterine wall nodule (2.8cm × 2.4cm) and a new hypoechoic nodule on the posterior uterine wall.
2025/5/25	After 42 days of amenorrhea, follow-up ultrasonography revealed masses in the anterior and posterior uterine walls, measuring 6.3cm × 5.6cm and 2.5cm × 1.6cm, respectively.
2025/5/26	Chest computed tomography showed multiple slightly hyperdense lesions in the thoracic skeleton.
2025/5/28	^18^F-FDG PET/CT demonstrated intense FDG uptake in the mass located in the posterior uterine wall, raising suspicion of malignancy.
2025/5/29	The patient experienced the onset of menstruation.
2025/6/9	After completion of menstruation, the patient underwent surgical enucleation of the uterine mass; histopathological examination confirmed a benign uterine leiomyoma.

## Discussion

In the present case, the primary indication for performing ^18^F-FDG PET/CT was to evaluate multiple skeletal lesions detected on computed tomography and to exclude metastatic disease. However, PET/CT unexpectedly revealed two uterine lesions with distinctly different metabolic patterns. Notably, the nodule located on the posterior uterine wall exhibited markedly intense FDG uptake, with an extremely high SUVmax that further increased on delayed imaging, suggesting a high probability of malignancy, such as lymphoma or uterine sarcoma; however, benign lesions, including cellular or atypical leiomyoma, leiomyoma with degeneration, and adenomyosis, could not be completely excluded. Definitive diagnosis requires further histopathological and immunohistochemical evaluation.

Previous studies have consistently shown that most uterine leiomyomas demonstrate absent or only mild FDG uptake, whereas malignant uterine tumors usually exhibit significantly increased glucose metabolism. Consequently, FDG PET/CT has long been considered a useful adjunctive tool for differentiating benign from malignant uterine masses. Some investigators have proposed SUVmax cutoff values to improve diagnostic accuracy. For example, an SUVmax threshold of 7.5 has been suggested in certain studies (7). However, there is considerable overlap in FDG uptake between benign and malignant uterine lesions, and such thresholds should not be interpreted as definitive diagnostic criteria. In the present case, the SUVmax of the posterior uterine lesion far exceeded this proposed threshold, highlighting the limitations of relying solely on quantitative FDG parameters. This finding indicates that even extremely high FDG uptake does not unequivocally indicate malignancy. Magnetic resonance imaging (MRI), particularly with diffusion-weighted imaging (DWI), has demonstrated high diagnostic performance in differentiating uterine sarcomas from leiomyomas. Previous studies have shown that features such as high DWI signal intensity relative to the endometrium, low apparent diffusion coefficient (ADC) values, presence of necrosis, and irregular tumor margins are suggestive of malignancy, whereas low T2 signal intensity and lower DWI signal are more indicative of benign leiomyomas (8–10). Moreover, ^18^F-FES PET/CT (11), and ^18^F-FLT PET (12) have also shown potential value in differentiating benign from malignant uterine tumors.

The underlying mechanisms responsible for increased FDG uptake in benign uterine leiomyomas remain incompletely understood. Potential contributing factors include estrogen levels, cellular expression of glucose transporters and hexokinase, cellular density within leiomyomas, blood flow perfusion, growth factor expression, and inflammatory processes (5). These factors may lead to increased glucose utilization and subsequent high FDG accumulation, thereby mimicking the imaging appearance of malignant tumors on PET/CT.

In this case, it is noteworthy that the patient experienced the onset of menstruation on the day following the PET/CT examination (day 45 after amenorrhea). Previous studies have reported that FDG uptake in uterine leiomyomas may vary with the menstrual cycle, and is often higher during the late luteal phase (13). In the present case, the PET/CT examination was performed exactly during the late luteal phase; therefore, we speculate that the increased FDG uptake in the uterine leiomyoma may be associated with both menstrual cycle irregularity and a specific phase of the menstrual cycle. However, a marked difference in metabolic activity was observed between the anterior and posterior uterine wall leiomyomas in this patient. Literature has suggested that in patients with multiple uterine leiomyomas, lesions with higher FDG uptake often show higher signal intensity on T2-weighted magnetic resonance imaging compared with those with lower FDG uptake (13). However, the mechanisms underlying the marked differences in FDG metabolism among uterine leiomyomas at different sites within the same patient remain unclear.

In conclusion, this case illustrates an important diagnostic pitfall in nuclear medicine practice: benign uterine leiomyomas may occasionally exhibit markedly increased FDG uptake and closely resemble malignant uterine tumors on imaging. Awareness of this phenomenon is essential to avoid misdiagnosis and unnecessary aggressive treatment, ultimately contributing to optimal patient management. At a recent telephone follow-up, the patient reported no discomfort and expressed satisfaction with the treatment process.

## Data Availability

The original contributions presented in the study are included in the article/Supplementary Material. Further inquiries can be directed to the corresponding authors.
